# Reshuffling of the Coral Microbiome during Dormancy

**DOI:** 10.1128/aem.01391-22

**Published:** 2022-11-16

**Authors:** Anya L. Brown, Koty Sharp, Amy Apprill

**Affiliations:** a Woods Hole Oceanographic Institution, Woods Hole, Massachusetts, USA; b Roger Williams University, Bristol, Rhode Island, USA; Georgia Institute of Technology

**Keywords:** *Astrangia poculata*, dormancy, nitrification, coral, microbiome, quiescence

## Abstract

Quiescence, or dormancy, is a response to stressful conditions in which an organism slows or halts physiological functioning. Although most species that undergo dormancy maintain complex microbiomes, there is little known about how dormancy influences and is influenced by the host’s microbiome, including in the temperate coral Astrangia poculata. Northern populations of *A. poculata* undergo winter quiescence. Here, we characterized wild *A. poculata* microbiomes in a high-resolution sampling time series before, during, and after quiescence using 16S rRNA gene sequencing on active (RNA) and present (DNA) microbiomes. We observed a restructuring of the coral microbiome during quiescence that persisted after reemergence. Upon entering quiescence, corals shed copiotrophic microbes, including putative pathogens, suggesting a removal of these taxa as corals cease normal functioning. During and after quiescence, bacteria and archaea associated with nitrification were enriched, suggesting that the quiescent microbiome may replace essential functions through supplying nitrate to corals and/or microbes. Overall, this study demonstrates that key microbial groups related to quiescence in *A. poculata* may play a role in the onset or emergence from dormancy and long-term regulation of the microbiome composition. The predictability of dormancy in *A. poculata* provides an ideal natural manipulation system to further identify factors that regulate host-microbial associations.

**IMPORTANCE** Using a high-resolution sampling time series, this study is the first to demonstrate a persistent microbial community shift with quiescence (dormancy) in a marine organism, the temperate coral *Astrangia poculata*. Furthermore, during this period of community turnover, there is a shedding of putative pathogens and copiotrophs and an enhancement of the ammonia-oxidizing bacteria (*Nitrosococcales*) and archaea (“*Candidatus* Nitrosopumilus”). Our results suggest that quiescence represents an important period during which the coral microbiome can reset, shedding opportunistic microbes and enriching for the reestablishment of beneficial associates, including those that may contribute nitrate while the coral animal is not actively feeding. We suggest that this work provides foundational understanding of the interplay of microbes and the host’s dormancy response in marine organisms.

## INTRODUCTION

Nearly all animal, plant, and bacterial phyla include species that undergo dormancy to survive periods of harsh environmental stress. Dormancy represents a resting state, in which metabolic functions are depressed (1, 2). In bacteria, dormancy ensures their persistence in hosts and is a trait of both pathogens and beneficial symbionts (3, 4). In general, dormancy can be composed of multiple phases: preparation (before dormancy begins), initiation (onset of dormancy), maintenance (metabolic suppression, depletion of energy stores), potentiation (beginning of the post-dormant periods), and activation (resumption of activity) (5).

Hosts that undergo dormancy are also involved in complex associations with microorganisms. Host-associated microbes are involved in host immunity, physiology, survival, and metabolic function and thus likely are influenced and can be influenced by dormancy. The role of microbes in host dormancy is an emerging field; thus, our understanding of the microbial role, and even our understanding of the microbial shifts surrounding dormant periods, are limited. Indeed, microbes may influence the onset and cessation of dormancy or replace host functioning during periods of dormancy.

In several hosts, including bears, squirrels, crickets, and parasitoid wasps (6–9), dormancy is associated with shifts in the composition of the host’s microbiome. One role these community shifts play may be to replace resource acquisition or use while host functioning is shut down or reduced (6–9). For example, in ground squirrels, the restructuring of the gut is mediated by food availability (6). During hibernation the gut microbiome plays an important role in nitrogen recycling while the squirrel is fasting (9). Dormant states are also associated with pathogen avoidance; for example, nematodes enter diapause to avoid infection (e.g., by not ingesting pathogens) (10).

In aquatic invertebrates, the onset of dormancy, or quiescence, is associated with harsh environmental conditions, such as winter (2). Few examples of dormancy are found in cnidarians, and even fewer in the class Anthozoa. However, the temperate scleractinian coral Astrangia poculata, is known to undergo quiescence in the winter months, which is thought to be a response to extreme cold temperatures (11). Similar to other species that undergo dormancy, quiescent *Astrangia poculata* have a distinct phenotype. They pull in their tentacles, form a puffed-up ring around their oral disc, do not respond to tactile stimulation, and do not actively feed. During quiescence there are also physiological shifts, including lowered coral colony growth rates (11), polyp loss (12), and shifts in the coral transcriptome, associated with thermal stress and lowered motility (13). Additionally, the physiological costs of dormancy can last beyond winter into spring (14).

*Astrangia poculata* represents a multidomain symbiosis, involving specific bacteria and archaea (15), and it engages in facultative symbiosis with the eukaryotic microalga Breviolum psygmophilum (family *Symbiodiniaceae*), the same genus of microalgae found in many tropical corals (16). This coral species shows two forms: a “white,” or aposymbiotic, phenotype and a “brown,” or symbiotic, phenotype, depending on the visible presence of microalgae in their otherwise transparent tissues. Although in symbiosis with photosynthetic algae, the coral mainly relies on heterotrophy for nutrition (14, 17).

*A. poculata* microbiomes are dominated by taxa similar to those of tropical corals at the class level (e.g., *Gamma*- and *Alphaproteobacteria*; *Cytophagia*, *Flavobacteria*), although the *A. poculata* microbiome is generally less diverse (15). As the similarities in taxa suggest, the microbiome of *Astrangia* also is expected to function similarly to those of tropical corals in nutrient cycling, sources of nutrition, immunity, and defense (18–22).

*A. poculata* microbiomes shift with season. The *A. poculata* winter microbiome is enriched in *Clostridiaceae*, *Flavobacteriaceae*, and *Rickettsiaceae* and lower in alpha diversity compared to the fall and spring microbiomes (15). In the spring, the microbiome alters in composition to a less variable microbial community compared to winter, fall, and summer (15). The shift in microbial communities from fall to spring also corresponds to tropical coral microbiomes that undergo cyclical mucus shedding (23). The seasonal shifts in *Astrangia* microbiomes are thought to be associated with quiescence; however, a detailed characterization of the microbial shifts that occur around quiescence is needed to determine how dormancy may impact the microbiome and vice-versa.

**FIG 1:**
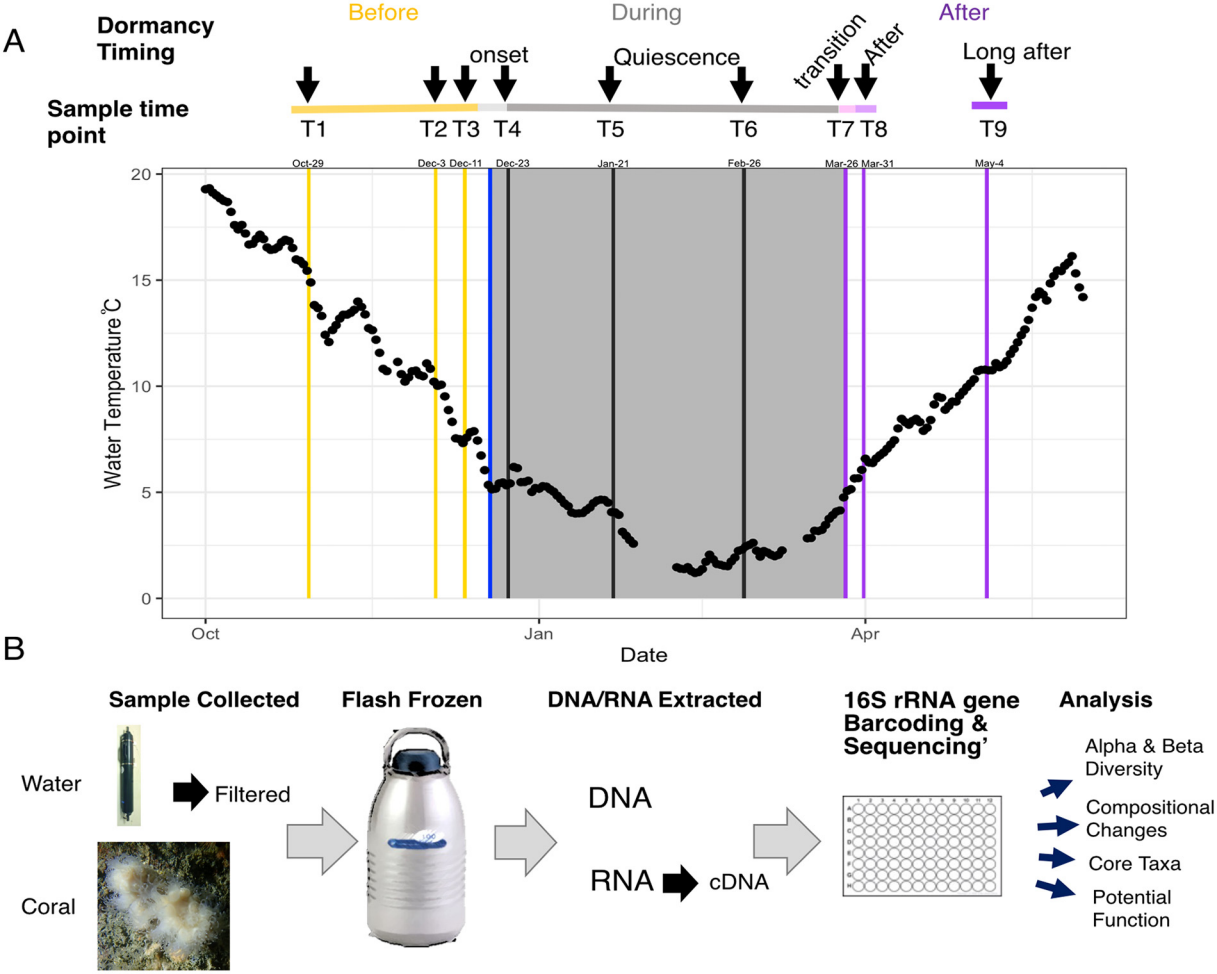
Overview of field experimental sampling, sample processing, and analysis. (A) Mean daily temperature based on station BZBM3 in Woods Hole, MA. Dates range from October 2020 to May 2021. The shaded region represents the time in which corals were in quiescence. Lines refer to sampling periods around dormancy: yellow indicates before; the blue line refers to when corals went into quiescence; gray lines refer to quiescence; purple lines refer to after quiescence. Above the plot, the line and labels refer to when samples were taken, the designation of sample points, and naming of the phases of dormancy. (B) Schematic of the sampling protocol for corals (*n* = 10 per time point) and water (*n* = 4 per time point) and analyses.

Here, we collected a high-resolution sampling time series to characterize the shift in microbiome diversity and community structure as *Astrangia poculata* corals go into, remain in, and come out of quiescence (Fig. 1). Based on the results of seasonal studies and other studies of animal dormancy, we expected a shift in community composition throughout quiescence, lowered diversity of microbes in the winter, and decreased variability among individual coral colonies as they emerged from quiescence. As some microbes may also be dormant as the coral host enters dormancy, we compared the active (RNA) and present (DNA) microbiome over time to understand which taxa are contributing to host-associated microbiome activity during host dormancy. Lastly, we propose new hypotheses about the taxonomic shifts, their functional significance, and the implications for the host throughout the phases of dormancy (before, during, and after).

## RESULTS

*Astrangia poculata* collections occurred via scuba diving in Woods Hole, MA, over a 6-month period and began in late October when seawater temperatures were 15.4°C. During this time the coral polyps were extended and presumed to be feeding and metabolically active (Table 1, Fig. 1A). Three collections (each of 10 colonies) occurred during this prequiescence period (time points *T*1 to -*T*3). Corals (at 16 m) were observed to be quiescent on 18 December (5°C) by divers who observed the area daily. At this time, polyps were retracted and presumed to be no longer feeding. On 23 December 2020, the first quiescent corals were collected (5°C; *T*4; *n *= 10), and collections continued during quiescence (*T*5 and *T*6, *n *= 10 colonies each). On 24 March (5°C; *T*7), some corals emerged from quiescence and some did not; five quiescent and five emerged corals were collected. By 31 March (*T*8), all corals had emerged from quiescence (*n* = 10), and collections continued for one additional postquiescence period (15 April; *T*9, *n* = 10). Macronutrients (NH_4_^+^, NO_2_, silicate, PO_4_^3–^) were lower throughout the period of quiescence than in time points before quiescence. Results are shown with the F statistic, and numerator and denominator degrees of freedom, as well as the *P* value (NH_4_^+^: F_3,28_ = 11.684, *P* < 0.001; NO_2_^−^: F_3,28_ = 58.77, *P* < 0.001; silicate: F_3,28_ = 31.41, *P* < 0.001; PO_4_^3−^: F_3,28_ = 20.38, *P* < 0.001; see Fig. S1 in the supplemental material). Total nitrogen (TN) and total organic carbon (TOC) were variable and did not differ significantly over the dormancy time periods (F_3,24_ = 1.87, *P* = 0.16; F_3,24_ = 1.07, *P* = 0.38; respectively), although TOC was qualitatively higher while corals were dormant (Fig. S1).

**TABLE 1:** Coral and seawater sampling data for different analyses

Date	Temp (°C)	Time point	Timing (NMDS)	Dormancy timing
29 October 2020	15.1	*T*1	Before	Before
3 December 2020	10.2	*T*2	Before	Before
11 December 2020	7.3	*T*3	Before	Before
23 December 2020	5.3	*T*4	Onset	During
21 January 2021	4.1	*T*5	During	During
26 February 2021	2.3	*T*6	During	During
26 March 2021	4.8	*T*7	Transition	During/after (depending on the coral)
31 March 2021	6.1	*T*8	After	After
4 May 2021	10.8	*T*9	Long after	After

To investigate the microbial community associated with the coral and at each time point, the 16S rRNA genes of bacteria and archaea were amplified from DNA (present microbiome) and cDNA (active) extracted from one polyp of each coral colony and sequenced. Bacterial and archaeal sequences were also obtained from seawater adjacent to the coral habitat (*n *= 4 per time point) using the same approach (Fig. 1B). Raw sequences can be found in the NCBI SRA database (BioProject PRJNA860933).

After quality filtering and removal of taxa associated with the controls (0.73% of all amplicon sequence variants [ASVs]) and chloroplasts and mitochondria (6.7% of unique ASVs), we retained 12,964,163 sequences (median, 30,029.5) across all 238 samples (coral and water) and 19,656 unique ASVs. Four cDNA coral samples from different time periods (*T*1, *T*3, *T*4, *T*5,) were removed because of low numbers of sequences (<1,000). Unique ASVs were examined per sample time, and in the coral present and active microbiomes, we observed 10,504 and 9,681 unique ASVs, respectively; in the water present and active microbiomes we observed 1,184 and 6,060 unique ASVs, respectively. After rarefying (only used in the Hill number D^0^ or richness analysis), there were 1,307 sequences/sample and 8,242 unique ASVs across the data set (water and coral).

### Alpha diversity.

**FIG 2:**
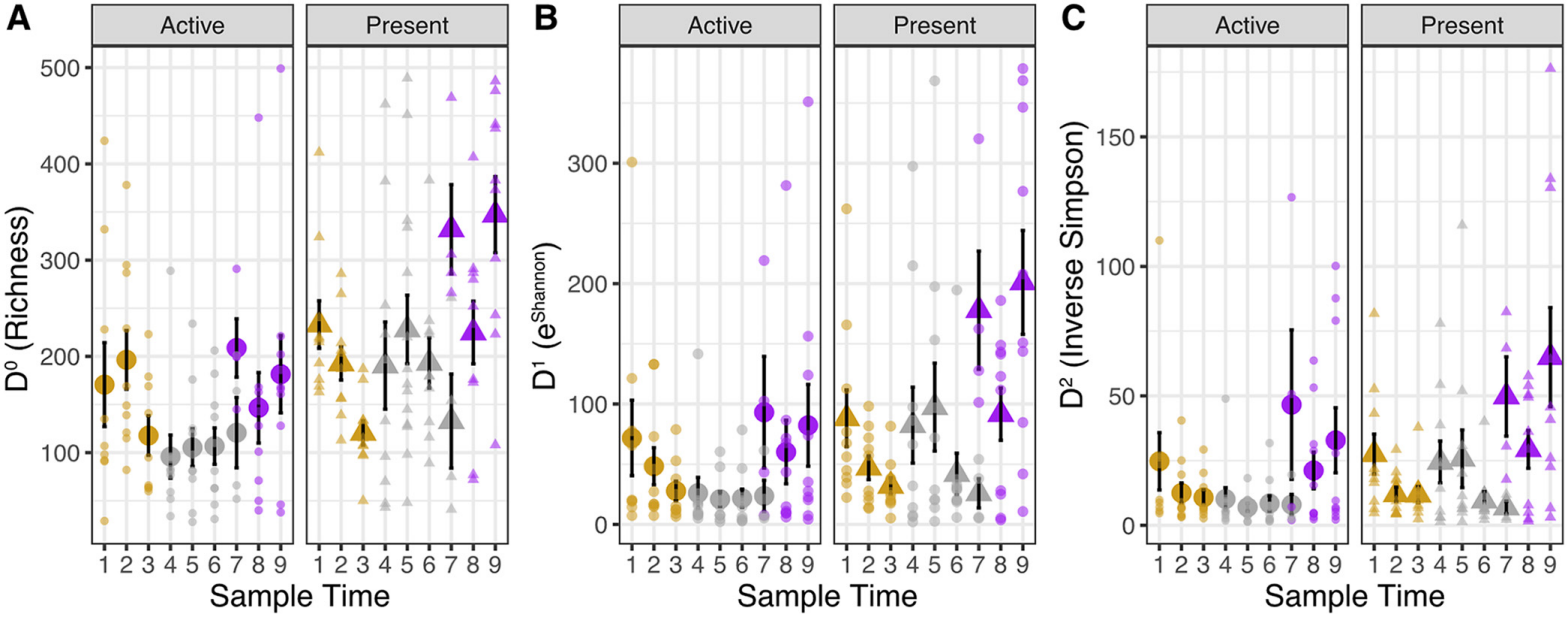
Coral microbiome alpha diversity decreases during quiescence. Plots show the mean ± standard error (SE) of alpha diversity measures. Colors indicate before (yellow), during (gray), and after (purple) quiescence. The active microbiome and present microbiome are separated by facets and by shape (circles and triangle, respectively). Smaller, transparent points represent raw values. (A) Hill D^0^ (rarefied richness); (B) D^1^ exponentiated Shannon diversity (not rarefied); (C) D^2^ or inverse Simpson diversity. D^0^ and D^1^ decreased during quiescence and then increased after quiescence in active microbes; however, in the present microbes, the diversity only changed (increased) after dormancy. Conversely, D^2^ remained low before and during quiescence and increased after quiescence for both DNA and cDNA.

As expected, there were fewer taxa in the active coral microbiome than in the present coral microbiome. This pattern was particularly evident in rarefied richness during and after dormancy (Fig. 2A, Table 2; active/present microbiomes, <0.05 for all diversity measures). Interestingly, for active microbiomes, we observed a significant decrease in alpha diversity as corals went into dormancy; it remained low as corals were in dormancy and then began to increase as corals exited dormancy (dormancy timing in D^0^ or richness: *P* = 0.004; D^1^ or exponentiated Shannon diversity: *P* = 0.002; active/present microbiomes, <0.05; Fig. 2A and B, Table 2). However, in the present microbiome, there were no significant differences in diversity between before and during dormancy, but similarly, we observed an increase after dormancy based on Tukey honestly significant difference (HSD) tests (in D^0^ and D^1^, Fig. 2A and B). Hill number D^2^ or the inverse Simpson index, the diversity measure influenced by dominance, significantly increased only after corals came out of quiescence in both the active and present microbiomes (dormancy timing: *P* = 0.002; Fig. 2C).

**TABLE 2:** Results of statistical analysis examined for diversity indices, from ANOVA analyses^*a*^

Measure	Treatment	Df	*F*	*P*
D^0^ (Richness)	Sampling time	7	2.5254	0.02
	Dormancy timing	1	8.38	0.004
	Active/present microbiome	1	26.48	<0.001
D^1^ (Exponentiated Shannon)	Sampling time	7	1.58	0.145
	Dormancy timing	1	10.22	0.002
	Active/present microbiome	1	16.64	<0.001
D^2^ (Inverse Simpson)	Sampling time	7	2.10	0.001
	Dormancy timing	1	9.58	0.002
	Active/present microbiome	1	5.1	0.02
Beta Dispersion	Sampling time	7	2.64	0.01
	Dormancy timing	1	1.00	0.32
	Active/present microbiome	1	0.597	0.44

a Df, degrees of freedom; *F*, frequency; F, F statistic; *P* < 0.05.

### Beta diversity.

**FIG 3:**
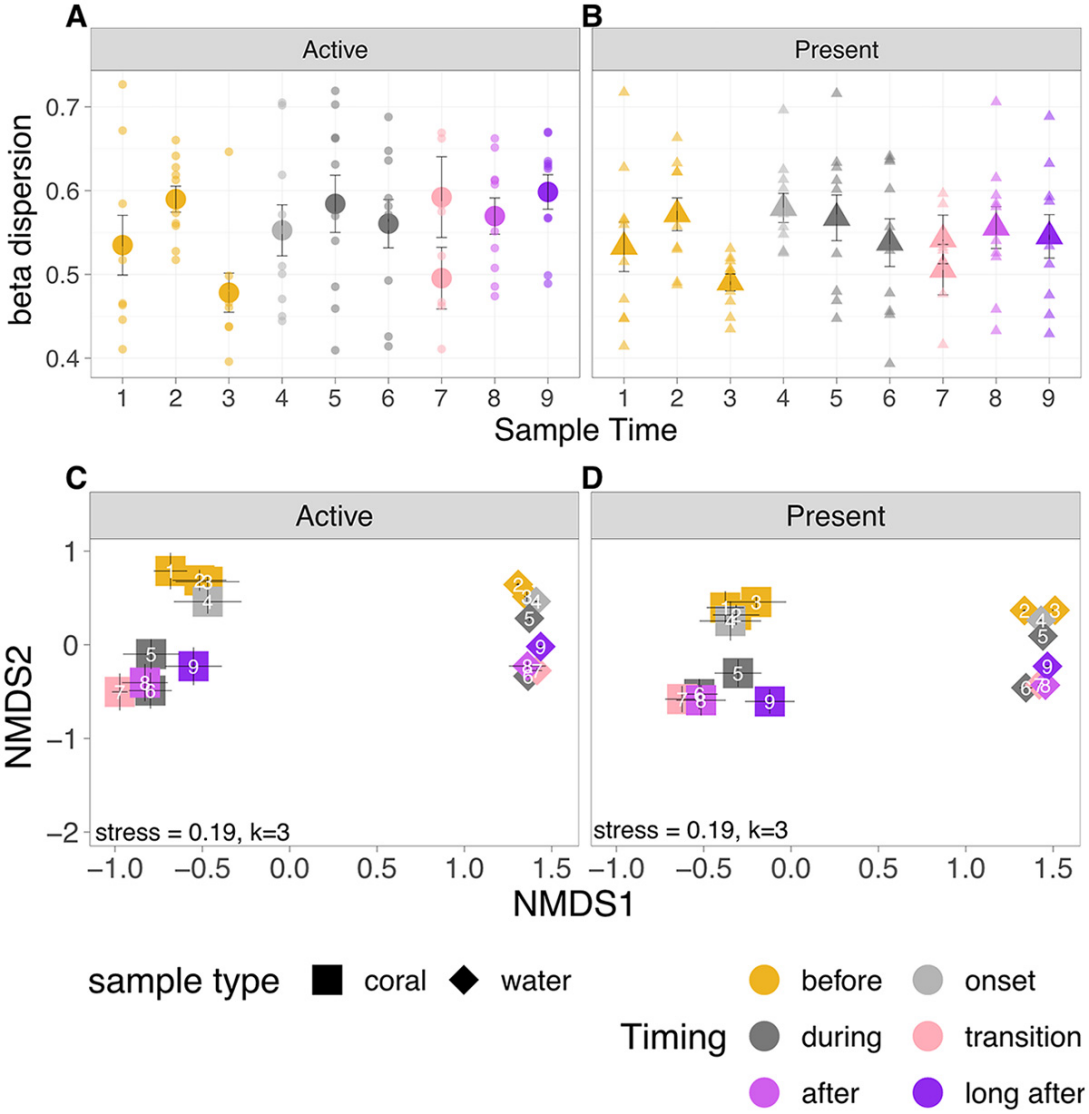
Coral microbiome beta diversity alters during quiescence. (A to D) The plots show mean ± SE beta dispersion of the (A) active and (B) present microbiomes for the coral (*n* = 9 to 10) and mean ± SE of the coral (*n* = 9 to 10) and water (*n* = 4) microbial communities in NMDS space in the (C) active and (D) present microbiomes. Colors represent timing, and shape indicates coral (squares) or water (diamonds) in the NMDS plot and active (circles) and present (triangles) in the dispersion plots. The numbers inside of the shapes (C and D) indicate the sampling time (see Table 1). Beta dispersion (A and B) was generally high but was significantly lower at time point 3, the sampling point before the onset of quiescence. Microbial community composition differed significantly (*P* < 0.05) based on timing, sampling time, and active/present microbiomes based on PERMANOVAs on the corals and the water (panels C and D).

Dispersion (beta diversity) was similar for both the active and present taxa (Fig. 3A and 3B, Table 2) and was consistent across the periods surrounding dormancy. However, the time point before corals went into quiescence (*T*3, 11 December 2020) showed significantly lower variability than that of all the other time periods, based on a Tukey HSD *post hoc* test (*P* < 0.05), and this was consistent in both the present and active microbiomes (Fig. 3A and B, Table 2; beta dispersion).

### Compositional shifts surrounding dormancy.

*Astrangia poculata* microbial community composition shifted significantly as corals went into quiescence and did not return to the same community after corals emerged from quiescence (Fig. 3c and D), suggesting a reshuffling of the microbiome that persisted even 2 months after corals were out of quiescence. Based on the permutational multivariate analysis of variance (PERMANOVA) analysis, we found significant effects of time (*R*^2^ = 0.05, *P* < 0.001), dormancy (before, during, and after: *R*^2^ = 0.08, *P* < 0.001), sample type (water/coral: *R*^2^ = 0.15, *P* < 0.001), and active/present microbiomes (DNA/cDNA: *R*^2^ = 0.02, *P* < 0.001).

Both the active (cDNA) and present (DNA) microbiomes changed in similar ways in nonmetric multidimensional scaling (NMDS) space (Fig. 3C and D), and we did not observe significant differences in microbial community composition within a time point between the active and present communities (Table S1; pairwise adonis results).

In both the active and present microbiomes, communities shifted markedly between before quiescence and during/after quiescence (time points 1 to 3 were different from time points 5 to 9) (Fig. 3C and D). The microbial community associated with the first time point for corals in quiescence (*T*4) was not significantly different from the time points before quiescence (*T*1 to *T*3) and from the time point 1 month later (*T*5), but it differed from all future time points (*T*6 to *T*9) (Fig. 3C and D; Table S1). Interestingly, postquiescent active microbiomes (time points 7, 8, and 9) did not significantly differ from corals in quiescence (time points 5 and 6) (Fig. 3C). The present microbiomes showed the same pattern, except that time point 9 (2 months after quiescence) was significantly different from the rest of the time points (Fig. 3D).

Seawater microbial community composition also changed over time significantly (*R*^2^ = 0.72, *P* = 0.001), and there were significant differences in active/present seawater microbiomes (*R*^2^ = 0.12, *P* = 0.001). The seawater microbiome was also consistently different from the coral microbiomes (Fig. 3C and D).

### Dormancy-associated taxon shifts.

**FIG 4:**
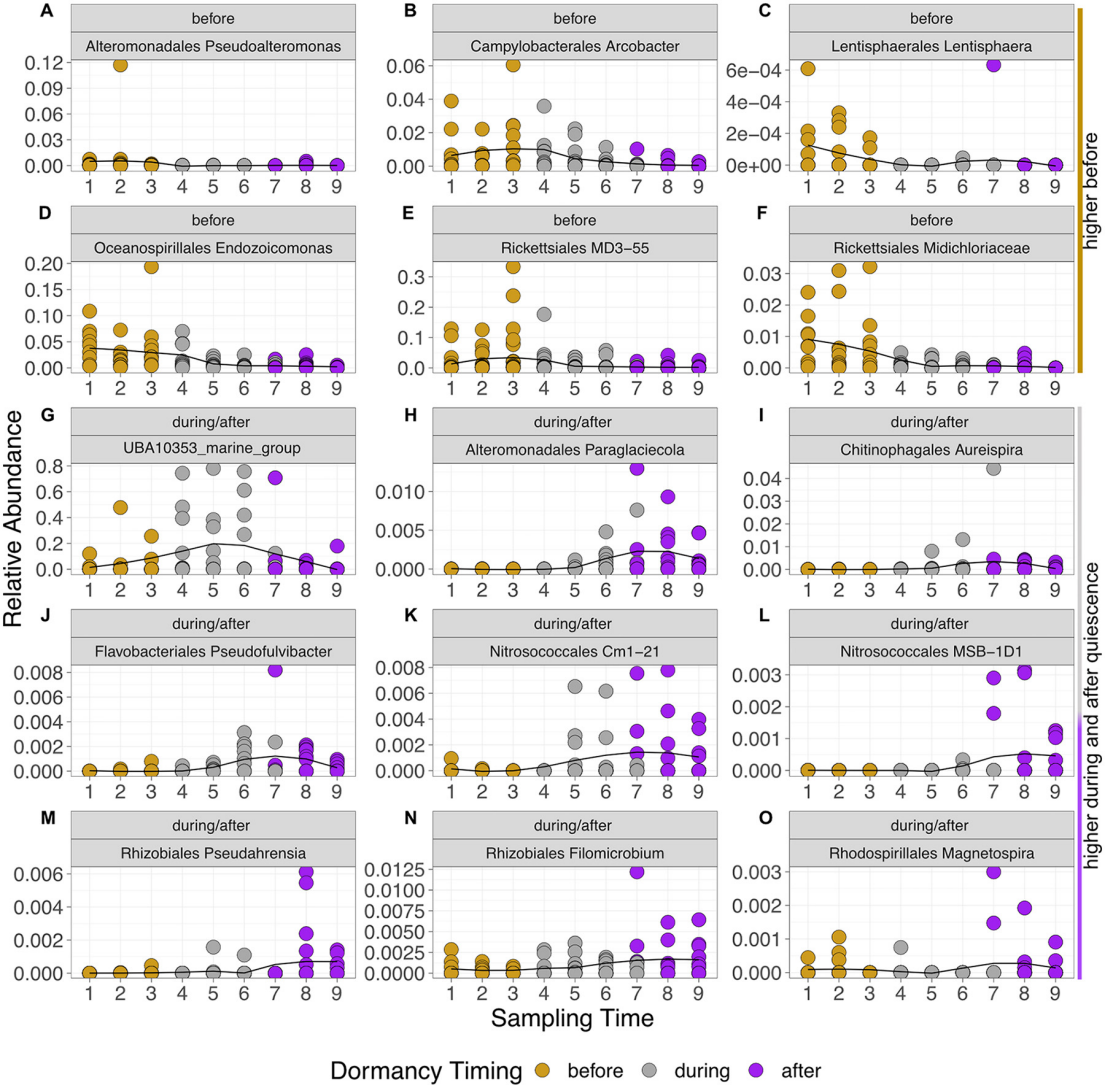
Relative abundance of selected active taxa (indicated by order and genus) that were significantly different according to the corncob analysis on the active microbiome-based ASVs. Points represent the relative abundance of ASVs in each coral (9 to 10 samples). Lines are a loess representation of the shape of the relationship based on the geom_smooth function in ggplot (in R). (A to G) Plots include taxa that were enriched before quiescence and at the first one or two time points during quiescence, (A) *Pseudoalteromonas* (*Alteromonadales*), (B) *Arcobacter* (*Campylobacterales*), (C) *Lentisphaera* (*Lentisphaerales*), (D) *Endozoicomonas* (*Oceanospirillales*), (E) MD3-55 (*Rickettsiales*), and (F) *Midichloriaceae*, and those that were enriched during quiescence, (G) UBA10353 marine group. (H to O) Lastly, those that were enriched as corals were midway through quiescence and continued to increase after quiescence, (H) *Paraglaciecola* (*Alteromonadales*), (I) *Aureispira* (*Chitinophagales*), (J) *Pseudofulvibacter* (*Flavobacteriales*), and (K) Cm1-21 (*Nitrosococcales*), or those that increase as corals come out of quiescence, (L) MSB-1D1 (*Nitrosococcales*), (M) *Pseudahrensia* (*Rhizobiales*), (N) *Filomicrobium* (*Rhizobiales*), and (O) *Magnetospira* (*Rhodospirillales*). Additional data are presented in Fig. S2.

A total of 61 ASVs were identified to change significantly during the phases of quiescence in the active microbiome (Fig. S2). Several taxa that were higher in abundance before corals went into quiescence began to wane at the beginning of quiescence (time points 4 and 5). These taxa included *Endozoicomonas*, *Arcobacter*, two groups of *Rickettsiales*, and *Pseudoalteromonas* (Fig. 4A to F). During quiescence, the UBA10353 marine group showed a marked increase that lasted throughout the quiescent period (time points 4 to 6, Fig. 4G). In late quiescence and as corals began to emerge (time points 5 to 9), several taxa were enriched, including those in the orders *Nitrosococcales* (Fig. 4K and L, Cm1-21 and MSB-1D1) and *Rhizobiales* (Fig. 4M and N), and the genus *Magnetospira* (Fig. 4O; for a full list, see Fig. S2).

Enrichment in the total present microbiome followed a similar pattern at the order level; however, it included more ASVs that shifted in abundance and presence (a total of 126, Fig. S2). In particular, bacteria in orders *Flavobacteriales*, *Chitinophagales*, *Cellvibrionales*, and *Sphingomonadales* were higher as corals emerged from quiescence and during quiescence, compared to before corals went into quiescence. Additionally, an ASV of “*Candidatus* Nitrosopumilus,” a taxon frequently associated with *A. poculata* (15, 24), was enriched during and after corals came out of quiescence.

Some of these taxonomic changes are likely temperature or environment driven, as the same taxa in the water column shifted similarly in abundance (e.g., *Synechococcus*, Fig. S2). However, most of the significant taxon shifts in the coral were not observed in the water column (Fig. S2).

### Core microbiome.

Across all time points, there were no taxa that were consistently present in the active microbiome of corals (100% of all samples), and only 5 ASVs were consistently present in 80% of samples across all time points (*Bacteroidea*, UBA4486, *Terasakiellaceae*, *Endozoicomonas*). Otherwise, core taxa changed by time point and varied between 4 and 8 ASVs within a time point, all of which were identified as significantly changing across dormancy time period in the corncob results (Fig. S3).

In the present microbiome, nine taxa were present in 80% of the samples (across all time periods). These taxa include those also found in the active microbiome and *Persicirhabdus*, *Pirellulaceae*, and *Rubripirellula*. Within a time point, core ASVs varied from 6 to 17 and included many that that changed significantly in the corncob results (Fig. S3).

We consistently observed two archaeal ASVs in the present core microbiome and in 65% of all active microbiomes which were associated with “*Candidatus* Nitrosopumilus” (Fig. S4). Only one of these ASVs was present in the water, and only in predormancy time points (Fig. S3). The other *Nitrosopumilus* ASVs we observed on the corals were in low relative abundances or not detectable in any seawater samples.

### Potential functional changes.

Of the 156 ASVs associated with significant shifts in the present and active microbiomes based on the corncob results, 106 taxa were assigned hypothesized functions from the FAPROTAX database. Based on the taxa that were identified as significantly enriched by the corncob results and their assignment of function with FAPROTAX, we found a reduction in the number of ASVs associated with photoautotrophy and photoheterotrophy on corals (in the cDNA and DNA) as coral went into and emerged from dormancy (Fig. S5A and B). We also observed a decline in the ASVs associated with intracellular parasites, nitrate reduction, sulfur/sulfite respiration, and methylotrophy in the coral active and present microbiomes as corals went into dormancy. ASVs associated with nitrogen fixation, nitrification, and dark sulfur oxidation increased after quiescence in the coral active microbiome.

## DISCUSSION

Here, we show evidence from a time series that encompassed a 3-month period of quiescence and cessation of feeding, which seasonally induces quiescence in the coral *Astrangia poculata*, is associated with a decrease in microbial diversity and a reshuffling of the coral’s microbial community. This alteration in the microbial community persists after corals emerge from their dormant state. In particular, taxa belonging to “*Ca*. Nitrosopumilus” and *Nitrosococcales*, groups of ammonia-oxidizing archaea and bacteria, respectively, and ASVs predicted to be associated with nitrification were enriched during and after quiescence. Copiotrophic bacteria, as well as a proposed pathogen of corals in the *Rickettsiales* order, decreased during quiescence. This study suggests that key microbial groups and potential functions are related to quiescence in *A. poculata*, which may play an important role in this yearly dormancy period and contribute to overall holobiont health and physiology.

### Streamlining of the active microbiome diversity during dormancy, but maintenance of variability.

Quiescence was associated with a streamlining of the coral’s microbiome. This was particularly evident in the active microbiome, as alpha diversity declined when corals entered dormancy. The loss of diversity is likely associated with the shedding of taxa associated with the dormancy period. Lowered alpha diversity is consistently a characteristic observed in dormant host-microbiome interactions, as diapausing parasitoid wasps (8) and ground squirrels also show a reduction in diversity of their dormant microbiomes (25).

As corals emerged from dormancy, we observed an increase in diversity (Hill numbers D^0^, D^1^, D^2^) in both the active and present microbiome. We expect that one of the drivers of this increase in alpha diversity postquiescence is the increase in feeding by the corals (11) and thus greater exposure to externally provisioned microbes. Emergence from quiescence could also be associated with increases in colonization of microbes from the water column, leading to the observed increases in alpha diversity. Interestingly, in time point 7, when 50% of the colonies had emerged from quiescence, the corals that had already emerged from quiescence had higher alpha diversity than the corals still in quiescence, suggesting that quiescence directly influences the observed changes in diversity. Further experimental work is needed to understand how quiescence emergence (e.g., mucus production, colonization from the water column) versus initiation of feeding influences the increase in diversity on corals emerging from quiescence.

Unexpectedly, coral microbiomes exhibited consistent levels of dispersion throughout the quiescence period in both the active and present microbial communities. This was surprising, as tropical corals, and other animal hosts exposed to stressors, often show increased variability during a stressor event (the Anna Karenina hypothesis [26, 27]). The lowered dispersion before quiescence is in contrast to data that had been previously collected seasonally, in which spring-collected (e.g., after quiescence) corals show decreased variability in their microbiomes relative to those collected during other seasonal time points (15) . We suggest that this difference could be due to variation in the locations sampled (Rhode Island versus Massachusetts), the lower resolution of sampling timing in the previous study, idiosyncratic differences in the environment associated with the day of sampling for the Rhode Island corals, or higher *B. psygmophilum* densities in the corals in previous studies. Previous research suggests that *B. psygmophilum* can influence microbiome beta dispersion after disturbance (laboratory antibiotic treatment) (24), so perhaps the near absence of *B. psygmophilum* across all of the corals in this study explains the consistent beta dispersion levels among the samples. Here, our sampling times encompassed multiple time points, including those in which some corals were in quiescence, and some were not (T7), 1 week after corals had emerged (*T*8) and 2 months later (*T*9), revealing that postquiescence is not always associated with lowered dispersion, and consistent levels of beta diversity (intercolony variability) may be an evolved trait of these corals.

### Reshuffling of the microbiome during dormancy.

Quiescence was associated with a reshuffling of the coral microbial community that persisted after the corals emerged from quiescence. In fact, few taxa were consistently associated with corals over the course of the sampling time because of the marked compositional shift that began during quiescence. The taxonomic shifts we observed before and after quiescence are similar to those previously documented in fall- and spring-collected corals (15). This concurrence suggests there may be predictable or cyclical patterns in the microbiome composition associated with dormancy timing that could be important for coral holobiont health. Additionally, reshuffling or shifts in the microbiome associated with dormancy are common among host-microbes, including diapaused copepods (28), parasitoid wasps (8), mosquitos (29), squirrels (6), and bears (7).

Among the hypotheses about the roles the microbiome plays in dormancy are (i) compositional shifts that lead to the removal of pathogens and/or (ii) the replacement or maintenance of critical functions (30). Here, we see evidence for these two hypotheses based on the identity of the taxa and predicted functions.

### Shedding of copiotrophs, including putative pathogens, during dormancy.

Among the taxon changes that are associated with community composition shifts are the loss of copiotrophic, and particularly, pathogen-associated, bacteria as corals undergo quiescence. These taxa include *Arcobacter*, Pseudomonas, and taxa in the order *Rickettsiales*, including a taxon with 97.6% sequence identity to tropical coral parasite, “*Ca.* Aquarickettsia rohweri” (31). Indeed, many of these taxa are associated with diseases in tropical corals (32–34). We also generally observed a decrease in copiotrophs, including *Endozoicomonas*, a putative beneficial symbiont in many hosts (35). In tropical corals, *Endozoicomonas* decreases in response to thermal stress (36), suggesting that it may be released when a host is stressed (e.g., an adaptive response) (37). Because *Endozoicomonas* tends to have large genomes (4 to 6 Mb) (38), they are likely energetically costly to maintain in symbiosis (39), which may be why they are reduced during dormancy (and other stressor events).

We suggest that the loss of copiotrophic bacteria and putative pathogens associated with the beginning of dormancy is potentially a mechanism or a consequence of a period with limited resources, when the holobiont cannot support energetically costly microbes. Thus, this loss is the result of either these microbes voluntarily or passively leaving the coral’s microbiome or an active ejection by the coral. Alternatively, a decline in *A. poculata* holobiont metabolism may trigger a concomitant decrease in the production of molecules that enrich for specific bacterial associates. Interestingly, the lone dormancy-only-associated microbe was most closely related to UBA10353, a bacterial group that produces pederin, a bioactive polyketide, in sponges (40). A resulting hypothesis is that the increase in this bacterium could result in production of antimicrobial compounds to reduce the colonization of microbes while the host is quiescent and/or help explain the loss of microbes associated with dormancy. In contrast with previous conclusions from seasonal characterization of *A. poculata* microbiomes (15), the higher-resolution sampling in this study reveals that putative pathogens are not higher in proportional abundances in winter months, as previously described (41) but, rather, are at their maximum just before entry into quiescence and are then shed after initiation of quiescence.

### Microbes involved in essential functioning during dormancy.

During dormancy we observed an increase and maintenance of microbes associated with ammonia oxidation, nitrification, and nitrogen fixation, suggesting that the microbiome plays a role in the maintenance and acquisition of nitrogen while corals are not actively feeding. Among the taxa that likely contribute to replacing host functions were archaea in the genus “*Ca*. Nitrosopumilus,” a known associate of *Astrangia poculata* (15, 24), and bacteria in the order *Nitrosococcales*, (Cm1-21, MSB-1D1).

“*Ca*. Nitrosopumilus” and *Nitrosococcales* are common ammonia oxidizers (42). Here, corals are not actively feeding and do not have any visible algal symbionts; thus, these ammonia oxidizers may play an important role in nitrate acquisition for the host or other essential microbes. Corals in late quiescence and early nonquiescence states also show increases in nitrate-reducing *Rhizobiales* (*Psuedahrensia* and *Filomicrobium*) (43) and *Magnetospira*, a likely nitrogen fixer (44). These taxa, along with the ammonia oxidizers, suggest that the microbial community likely continues to bolster nitrogen cycling in the host as corals emerge from quiescence and may help build energetic reserves that were depleted during quiescence (14). The presence of these bacteria and archaea, particularly the ammonia oxidizers, during dormancy and after dormancy may help explain some of the acquisition of nitrogen (i.e., ammonia, the host’s preferred dissolved inorganic Nitrogen, DIN, source) for the host in general, which is usually attributed to heterotrophy and enhanced by algal symbionts (14, 45). For example, the increase in nitrifying microbes may explain the higher δ^15^N values in *A. poculata* tissues previously found in the winter compared to the fall (14), as winter corals are quiescent and do not rely on heterotrophy (or photoautrophy). The nutrients in the water column (lowered during the winter months) also suggest that external provisioning is less likely at this time, increasing the importance of the potential microbial contribution to nutrient cycling. More research is needed to understand the role of the microbiome in the cycling of nitrogen in coral tissues and how this may impact coral fitness after quiescence.

Corals in late quiescence and after emergence also showed increases in *Flavobacteriales* (e.g., *Pseudofulvibacter*, *Ulvibacter*; Fig. 4, Fig. S2 and S5). As microbial heterotrophs, it is possible that these taxa play a role in carbon cycling on and/or with the host before and just as the coral begins to feed actively. For example, in sponges with high heterotrophic microbial loads, evidence suggests that microbes play a role in dissolved organic matter (DOM) assimilation (46). Alternatively, the increase in *Flavobacteriales* may be due to increases in food availability and is not necessarily host-associated (47).

Replacement of host nutrition during dormancy is a common theme in host-microbial systems. In ground squirrels (Ictidomys tridecemlineatus), the gut microbiome plays a critical role during hibernation to recycle nitrogen (from urea), which supports tissue growth while the animal is not feeding (9), which is evolutionarily advantageous leading up to the breeding season. Furthermore, diapausing *Daphnia* eggs are enriched in *Nitrospira* bacteria, suggesting that nitrification may be a function of dormant microbiomes in other hosts (48). In parasitoid wasps, microbiomes are responsible for synthesizing glucose for nutrition during dormancy (8). In both mosquitos and bears, microbes are suggested to also play a role in host provisioning and lipid storage (7, 29). Nutritional provisioning, particularly of nitrogen, during dormancy is potentially a convergent trait across host and microbes that undergo dormant periods.

### Onset of and emergence from quiescence timing.

Predictable, seasonal quiescence in *A. poculata* has been observed and documented in the field (11), and in the lab, quiescence can be experimentally triggered by lowering temperatures to 5°C (Sean Grace, personal communication; 13). Emergence from dormancy was similarly found by raising temperatures above 5°C (13). Here, our findings support that the onset of dormancy was associated with temperatures reaching 5°C, suggesting winter temperature as one of the environmental triggers for the onset of dormancy. Indeed, corals experienced temperatures as low as 2°C (Fig. 1). Because temperature appears to play a role in dormancy and microbial dynamics, as oceans warm, there likely will be consequences for the timing and duration of dormancy in northern populations of *A. poculata*, the microbial shifts surrounding dormancy, and coral host physiology. However, absolute temperature is likely not the only trigger for emergence: corals began to emerge from dormancy while water temperatures remained close to 5°C (Fig. 1, Table 1); thus, there may be other factors that lead to the cessation of quiescence. An additional hypothesis is that nutrient availability may influence dormancy. Nutrients (e.g., ammonia, phosphate) are lower during the winter. As the microbiome shifts swiftly during quiescence, it is possible there is an interplay between the environment, the host, and the microbiome that triggers the onset of and emergence from quiescence. Further experimental work, including isolating the effects of temperature and season, and characterizing the mucus metabolome throughout seasonal and environmental shifts, will help to elucidate the relationship between microbial shifts and quiescence. Because *A. poculata* are facultatively symbiotic with microalgae and can also be used in aquarium-based studies (24), it is an ideal marine experimental system for investigation of the role of the microbiome in animal host dormancy.

### Conclusions.

**FIG 5:**
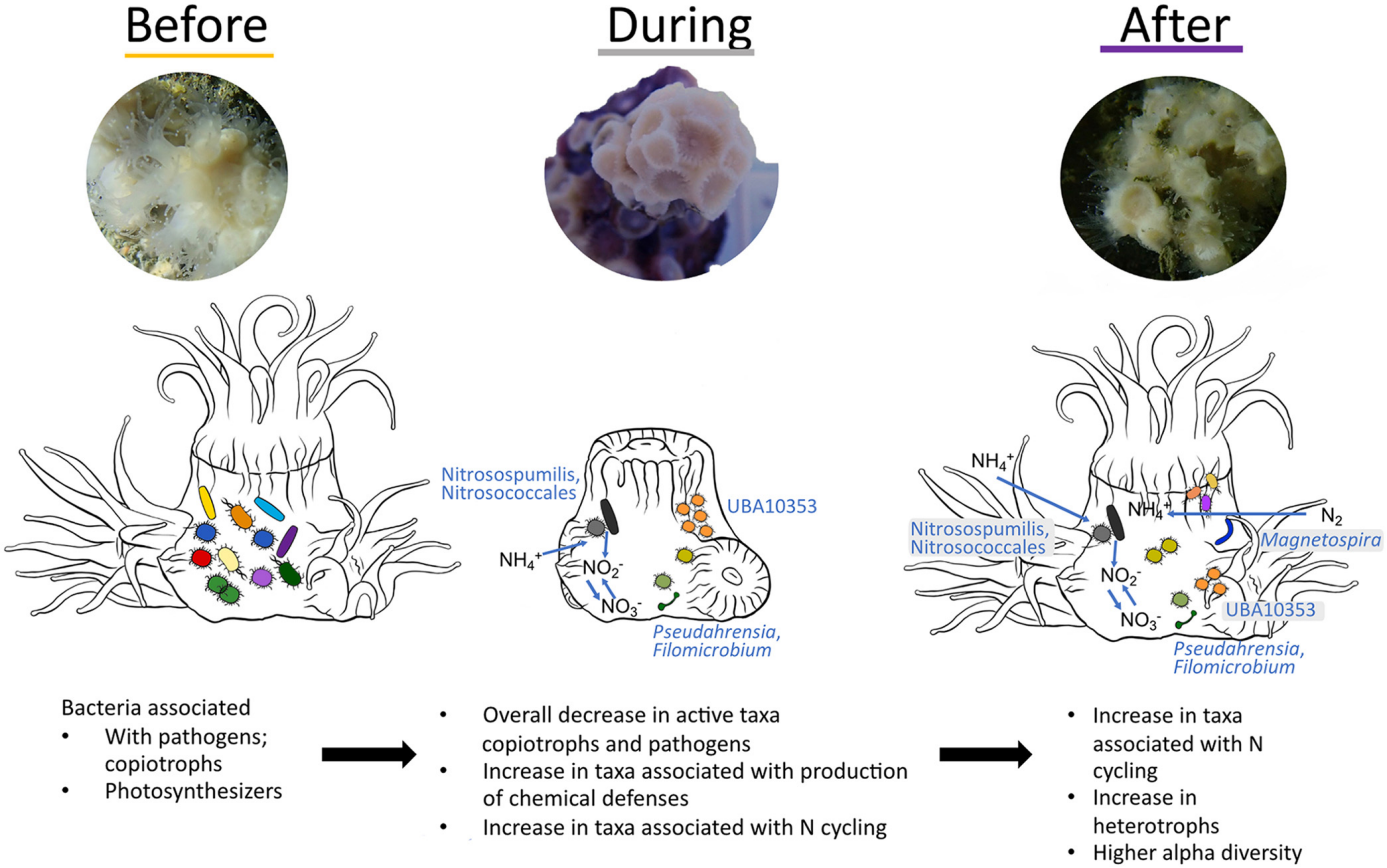
Conceptual diagram of the diversity and community shifts that occurred in the *Astrangia poculata* microbiome before, during, and after quiescence. Illustrations by Alicia Schickle.

Our findings suggest that *A. poculata* quiescence is involved in reshuffling the microbiome, leading to a new, persistent microbiome community structure. This shuffling is associated with shedding of potential pathogens, such as *Rickettsiales*, and a shift in the taxa that likely replace nutrition, such as “*Ca.* Nitrosopumilus,” while the host is inactive (Fig. 5). Overall, this study demonstrates that key microbial groups are related to quiescence in *A. poculata* and may play an indirect or direct role in the onset of and emergence from dormancy. Further understanding of the interactions between the coral and these specific microorganisms during this change in coral metabolic status will advance our understanding of coral host-microbiome dynamics and dormancy of host and microbes in general.

## MATERIALS AND METHODS

### Sample collection.

From late October 2020 to May 2021 we collected distinct white, or aposymbiotic, colonies of *A. poculata* at each of nine time points (*n* = 10 per time point; Table 1, Fig. 1). Corals were collected on SCUBA at 18 m from pilings on the Woods Hole Oceanographic Institution’s Iselin dock (41°31′25.1″N 70°40′19.3″W). We selected aposymbiotic colonies to reduce the potential effects of the algal symbionts in our understanding of dormancy-related microbial shifts. Selected colonies showed no visible coloration in any of the polyps (or near absence of algae; Fig. 1). Corals were collected using a hammer and chisel and were frozen in liquid nitrogen vapors immediately upon surfacing from the dive.

During eight of the time periods (starting on 3 December), we collected water samples in a 5-L Niskin bottle triggered at depth (18 m). We subsampled this water for macronutrients (25 mL, frozen to −20°C), which were analyzed as described previously (49). We also collected samples for total organic carbon and total nitrogen (40 mL acidified with concentrated phosphoric acid, 75 μL; *n* = 4 per time point, except time point 3, in which *n* = 3). For seawater microbial community analysis, we filtered 500 mL of the collected water through a 0.22-μm Sterivex filter (Millipore Sigma, Burlington, MA; *n* = 4 per time point) using peristaltic pressure and then flash froze the filter in liquid nitrogen vapors.

### Temperature data.

Seawater temperature data were from station BZBM3 in Woods, Hole, MA, measured 1.7 m from the mean lower low water line (50). Temperature data were averaged by day over the time period of sampling (October to May).

### Nucleic acid extractions and cDNA synthesis.

For both coral and water samples, DNA and RNA were extracted using the Quick DNA/RNA mini prep plus kit with ZR BashingBeads (0.1 and 0.5 mm; Zymo Research, Carlsbad, CA). For coral samples, one polyp (including mucus, tissue, and skeleton) of each coral colony was removed with a sterilized chisel and hammer. Coral fragments were added to the bead tubes and then suspended in 800 μL of DNA/RNA Shield and bead-beaten for 10 min at top speed on a vortexer. We added proteinase K (15 μL of 20 mg μL^−1^) to further break down cells and isolate the DNA and then continued with the rest of the steps in the manufacturer’s protocol, including the DNase step for the RNA portion of the sample. Extracted RNA was frozen at −80°C, and DNA was frozen in at −20°C until further analysis.

For water samples, we opened the plastic case of the Sterivex filter using a sterilized steel cutting implement, removed the filter using a sterilized razor, cut the filter into strips over a sterile petri dish, and placed the filter into the bead tube using sterile tweezers. We then added 1,000 μL of DNA/RNA Shield to the sample and bead tube. Then, 30 μL of proteinase K (20 mg μL^−1^) was added before we continued with the manufacturer’s protocol. Extraction blanks, which included reagents but no samples (*n* = 3 for the water protocol, *n* = 6 for the coral protocol), were carried out as well for both RNA and DNA.

We converted RNA to cDNA for sequencing the active microbiome using the New England Biolabs (Ipswich, MA) ProtoScript II first-strand cDNA synthesis kit. We followed the standard protocol and used 2 μL of coral RNA and 6 μL of water RNA as the template.

### 16S rRNA gene library prep.

We prepared DNA and cDNA for 16S rRNA gene sequencing of the V4 region using barcoded 515FY (51) and 806RB (52) primers that target bacteria and archaea with standard barcodes (53). The PCRs (25 μL) were performed in duplicate per sample and prepared using the high-fidelity (HF) Phusion master mix with HF buffer (12.5 μL/sample), dimethyl sulfoxide (DMSO; 0.75 μL/sample) (New England Biolabs), molecular-grade water (7.25 μL/sample), the primers (1.25 μL of each), and 1 μL of template. The thermocycler conditions were an initial denaturation step of 95°C for 2 mins, and then 30 cycles of 95°C (20s), 55°C (15s), and 72°C (5 min), and a final elongation step of 72°C for 10 min.

Each PCR was run on 1.5% agarose, and the correct band (determined by the location of the positive control, ~400 bp) was excised. Excised bands were extracted and purified with the MinElute gel purification kit (Qiagen, Inc., Germantown, MD). Purified PCR products were quantified with a Qubit device, diluted to 1 ng μL^−1^, and then pooled at 5 ng of purified product per sample. Each pool contained negative PCR controls with no visible bands and a mock community (Even, low community B; BEI Resources). The pools (3 total) were sequenced on an Illumina MiSeq instrument with 250-bp paired-end sequencing.

### Bioinformatics.

With the demultiplexed forward and reverse sequences, we used the DADA2 pipeline (54) in R (55) for quality control, merging sequences, and assigning amplicon sequence variants (ASVs). Forward and reverse reads were visually inspected for quality with DADA2 and ggplot2 and to determine the cutoff values (the average number of base pairs of which quality scores fell below 30) in the filter and trim step with the following parameters: filterAndTrim(fnFs, filtFs, fnRs, filtRs, truncLen = c(240, 150), maxN = 0, maxEE = c(2), rm.phix = TRUE, compress = TRUE, multithread = TRUE). Error rates were computed and used for sequence inference in DADA2. Sequences were then merged, and ASV tables were created. Because of the size of the data set, error rates and ASV tables were created per MiSeq run, the tables were then merged, and chimeras were checked and removed. Taxonomy was assigned using the SILVA v132 training set (56, 57), and retrieval of taxa from mock communities was checked.

The taxon table, ASV table, and metadata table were loaded into phyloseq (58), where chloroplasts and mitochondria were removed. Using the decontam package (59), we removed contaminant taxa using the prevalence of taxa (at 0.01) in the negative controls (including PCR negatives, extraction kit blanks, and water filter blanks for both DNA and cDNA).

Alpha diversity was calculated using Hill numbers D^0^, D^1^, and D^2^, which correspond to richness (rarefied), exponentiated Shannon diversity, and the inverse Simpson index, respectively (60). Higher D values indicate more even and speciose communities. We estimated diversity indices using phyloseq.

To compare the variability in coral communities over time, we computed Bray Curtis dissimilarities on the relative abundances of taxa within a sample. We then quantified the distance from each sample point to the group’s centroid (beta dispersion) using the betadisper function in the vegan package (61).

We tested for significant differences in alpha diversity and dispersion using linear models, comparing dormancy timing (before, during, after), sample time (time points 1 to 9), and active/present microbiome (cDNA/DNA) in R (54). Residuals were visually inspected to meet assumptions of heteroscedasticity and normality. Significance was assessed using ANOVA from the car package (62). When necessary, *post hoc* tests (Tukey’s HSD) were used to evaluate differences among levels in treatments.

To examine compositional changes within *Astrangia poculata*’s microbiome throughout the phases of dormancy (before, at the onset, during, and after), we used Bray Curtis dissimilarity matrices based on relative abundance. We then compared the microbial community composition using PERMANOVA in the vegan package (61) with sample times (1 to 9) and the timing around dormancy (before, during, after) and active/present microbiome as factors. To understand the differences in community composition across time points and types of sample, we used pairwise comparisons with the EcolUtils package (63). Coral and water microbial communities were visualized on an NMDS plot.

We assessed which microbial taxa changed in relative abundance with respect to timing of dormancy (before versus during, before versus after) using corncob (64). This method uses a beta-binomial model on the counts (number of reads) for each ASV to determine which taxa are significantly enriched from a reference level (in this case, before dormancy) in different treatments and compares them iteratively. We compared the active (DNA) and present (RNA) microbiomes from the coral and water separately.

To determine which microbial taxa compose the core of *Astrangia poculata*, and how these taxa changed over time, we determined which taxa were present at 80% prevalence across all samples within a time point (18) with the microbiome package (65).

We hypothesized potential functions of the microbiome using a functional inference tool based on taxonomy. Although there are drawbacks to tools that predict function from taxonomy (66, 67), here, we took a conservative approach and used broad categorizations of functions using the FAPROTAX database (68) and microeco package (69) in R, which were generated from published metabolic and ecological functions and suggested for environmental data (70). We extracted the functions associated with ASVs that were determined to be significantly enriched based on the corncob analysis to understand potential functional shifts across the dormancy time periods in the active and present microbiomes.

### Data availability.

Sequences are available on the NCBI Sequence Read Archive (SRA) BioProject PRJNA860933 under the accession numbers: SAMN29871893-SAMN29872136.
