# Supplementary material for: Reshuffling of the Coral Microbiome during Dormancy
Source: Appl Environ Microbiol. 2022 Nov 16;88(23):e01391-22. doi: 10.1128/aem.01391-22 (PMC9746315; doi:10.1128/aem.01391-22)
Supplement: Supplemental file 1 — Supplemental material. Download aem.01391-22-s0001.pdf, PDF file, 2.0 MB [file aem.01391-22-s0001.pdf]

Supplemental Figures

Reshuffling of the coral microbiome during dormancy

Anya L Brown[1,2*], Koty Sharp[3], Amy Apprill[1]

[1]Woods Hole Oceanographic Institution, Woods Hole, MA
[2]Bodega Marine Lab, UC Davis, Bodega Bay, CA (present address)
[3]Roger Williams University, Bristol, RI
*corresponding author email: anybrown@ucdavis.edu

Figure Legends

Figure S1. Raw data and boxplot of nutrient concentrations for A) NH_4^+, B) NO_2, C) Silicate, D) PO_4^{3-}, E) Total Nitrogen (TN), and F) Total organic carbon (TOC). Macronutrient concentrations were low throughout dormancy (NH_4^+: $F_{3, 28} = 11.684$, $p < 0.001$; NO_2: $F_{3, 28} = 58.77$, $p < 0.001$; silicate: $F_{3, 28} = 31.41$, $p < 0.001$; PO_4^3: $F_{3, 28} = 20.38$, $p < 0.001$). Total Organic Carbon and Total Nitrogen were variable in the winter, and did not differ significantly depending on dormancy timing for ($F_{3,24} = 1.87$, $p = 0.16$; $F_{3,24} = 1.07$, $p = 0.38$).

Figure S2. Differential abundance of ASVs (each point) Before vs During and Before vs After quiescence in the coral (active and present) and water (active and present). Values are effect size ± se output from the corncob model for each ASV. Points are colored based on whether they were enriched before (coral: yellow, water: light blue), during (coral: gray, water: blue), and after (coral: purple, water: dark blue).

Figure S3. Relative abundance of ASVs associated with the core microbiome (at 80% prevalence) of the cDNA and DNA. Each facet is labeled by Order and Genus. Points are colored by sample type (yellow indicates coral, blue indicates water) and present/active microbiomes

(lighter colors represent the active microbiome and darker colors represent the present). Lines are created by the loess function in ggplot2.

Figure S4. Relative abundance across all replicates in a time points of ASVs in the archaeon genus *Ca.* Nitrosopumilus in the coral (A) active, (b) present microbiomes and the water (c) active and (d) present microbiomes for each sampling point. Each color within the bar represents a different ASV associated with the DNA core (at 80% prevalence) or cDNA core (at prevalence 65%), represented by the asterisk (ASV 2,3) or ASVs that were significantly different based on the corncob results represented by ‡ (ASV 1 and 2). ASV 2 was significantly differentially abundant in the DNA based on the corncob results and is part of the coral core community (Fig S2).

Figure S5. Potential functions based on the FAPROTAX database (Liang *et al.*, 2020) assigned to the ASVs (here represented at the genus level) that are significantly changing based on the corncob results. A point represents that the function is present in that genus in the (A) present microbiome (i) before versus after corals are in quiescence and (ii) before versus during corals are in quiescence; and (B) in the active microbiome (i) before versus after corals are in quiescence and (ii) before versus during corals are in quiescence. Colors represent if the taxa/function are enriched before, during or after quiescence.

Figure S1

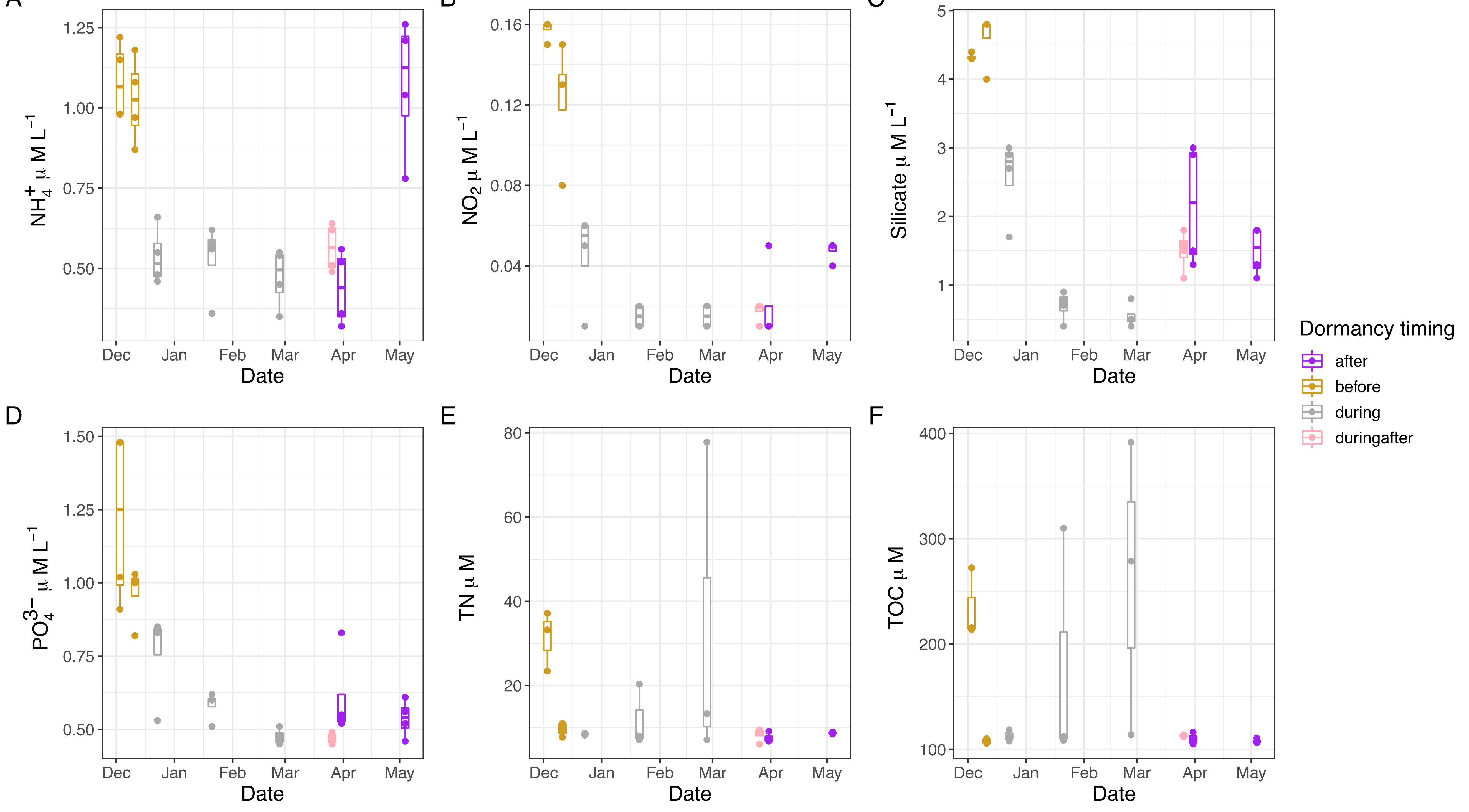

A
B
C
D
E
F
NH4+ μ M L−1
NO2 μ M L−1
Silicate μ M L−1
PO43− μ M L−1
TN μ M
TOC μ M
Date
Dec
Jan
Feb
Mar
Apr
May
Dormancy timing
after
before
during
duringafter

Figure S2

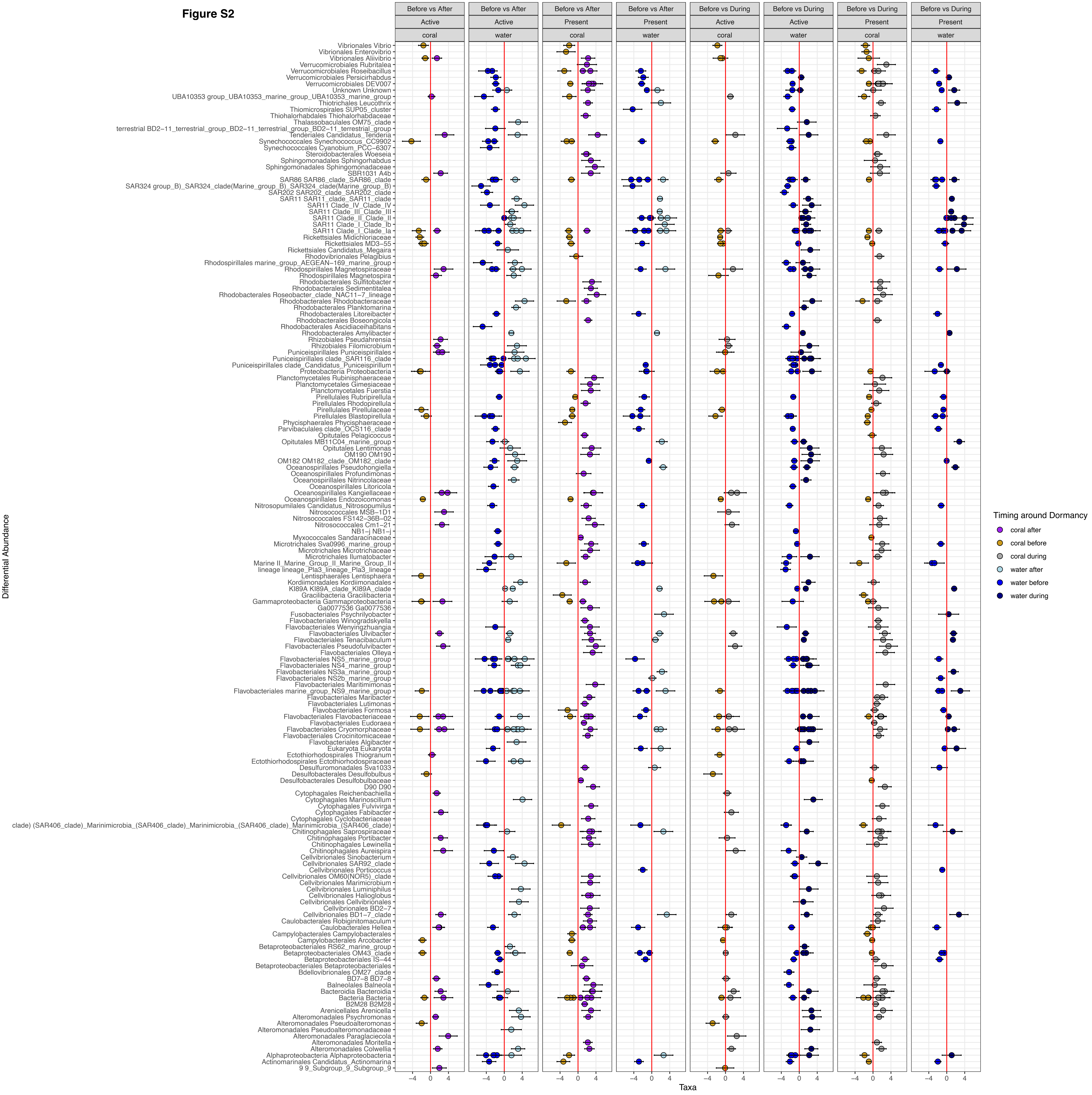

Before vs After
Active
coral
Before vs After
Active
water
Before vs After
Present
coral
Before vs After
Present
water
Before vs During
Active
coral
Before vs During
Active
water
Before vs During
Present
coral
Before vs During
Present
water
Vibrionales Vibrio
Vibrionales Enterovibrio
Vibrionales Aliivibrio
Verrucomicrobiales Rubritalea
Verrucomicrobiales Roseibacillus
Verrucomicrobiales Persicirhabdus
Verrucomicrobiales DEV007
Unknown Unknown
UBA10353 group_UBA10353_marine_group_UBA10353_marine_group
Thiotrichales Leucothrix
Thiomicrospirales SUP05_cluster
Thiohalorhabdales Thiohalorhabdaceae
Thalassobaculales OM75_clade
terrestrial BD2−11_terrestrial_group_BD2−11_terrestrial_group
Tenderiales Candidatus_Tenderia
Synechococcales Synechococcus_CC9902
Synechococcales Cyanobium_PCC−6307
Steroidobacterales Woeseia
Sphingomonadales Sphingorhabdus
Sphingomonadales Sphingomonadaceae
SBR1031 A4b
SAR86 SAR86_clade_SAR86_clade
SAR324 group_B)_SAR324_clade(Marine_group_B)_SAR324_clade(Marine_group_B)
SAR202 SAR202_clade_SAR202_clade
SAR11 SAR11_clade_SAR11_clade
SAR11 Clade_IV_Clade_IV
SAR11 Clade_III_Clade_III
SAR11 Clade_II_Clade_II
SAR11 Clade_I_Clade_Ib
SAR11 Clade_I_Clade_Ia
Rickettsiales Midichloriaceae
Rickettsiales MD3−55
Rickettsiales Candidatus_Megaira
Rhodovibrionales Pelagibius
Rhodospirillales marine_group_AEGEAN−169_marine_group
Rhodospirillales Magnetospiraceae
Rhodospirillales Magnetospira
Rhodobacterales Sulfitobacter
Rhodobacterales Sedimentitalea
Rhodobacterales Roseobacter_clade_NAC11−7_lineage
Rhodobacterales Rhodobacteraceae
Rhodobacterales Planktomarina
Rhodobacterales Litoreibacter
Rhodobacterales Boseongicola
Rhodobacterales Ascidiaceihabitans
Rhodobacterales Amylibacter
Rhizobiales Pseudahrensia
Rhizobiales Filomicrobium
Puniceispirillales Puniceispirillales
Puniceispirillales clade_SAR116_clade
Puniceispirillales clade_Candidatus_Puniceispirillum
Proteobacteria Proteobacteria
Planctomycetales Rubinisphaeraceae
Planctomycetales Gimesiaceae
Planctomycetales Fuerstia
Pirellulales Rubripirellula
Pirellulales Rhodopirellula
Pirellulales Pirellulaceae
Pirellulales Blastopirellula
Phycisphaerales Phycisphaeraceae
Parvibaculales clade_OCS116_clade
Opitutales Pelagicoccus
Opitutales MB11C04_marine_group
Opitutales Lentimonas
OM190 OM190
OM182 OM182_clade_OM182_clade
Oceanospirillales Pseudohongiella
Oceanospirillales Profundimonas
Oceanospirillales Nitrincolaceae
Oceanospirillales Litoricola
Oceanospirillales Kangiellaceae
Oceanospirillales Endozoicomonas
Nitrosopumilales Candidatus_Nitrosopumilus
Nitrosococcales MSB−1D1
Nitrosococcales FS142−36B−02
Nitrosococcales Cm1−21
NB1−j NB1−j
Myxococcales Sandaracinaceae
Microtrichales Sva0996_marine_group
Microtrichales Microtrichaceae
Microtrichales Ilumatobacter
Marine II_Marine_Group_II_Marine_Group_II
lineage lineage_Pla3_lineage_Pla3_lineage
Lentisphaerales Lentisphaera
Kordiimonadales Kordiimonadales
KI89A KI89A_clade_KI89A_clade
Gracilibacteria Gracilibacteria
Gammaproteobacteria Gammaproteobacteria
Ga0077536 Ga0077536
Fusobacteriales Psychrilyobacter
Flavobacteriales Winogradskyella
Flavobacteriales Wenyingzhuangia
Flavobacteriales Ulvibacter
Flavobacteriales Tenacibaculum
Flavobacteriales Pseudofulvibacter
Flavobacteriales Olleya
Flavobacteriales NS5_marine_group
Flavobacteriales NS4_marine_group
Flavobacteriales NS3a_marine_group
Flavobacteriales NS2b_marine_group
Flavobacteriales Maritimimonas
Flavobacteriales marine_group_NS9_marine_group
Flavobacteriales Maribacter
Flavobacteriales Lutimonas
Flavobacteriales Formosa
Flavobacteriales Flavobacteriaceae
Flavobacteriales Eudoraea
Flavobacteriales Cryomorphaceae
Flavobacteriales Crocinitomicaceae
Flavobacteriales Algibacter
Eukaryota Eukaryota
Ectothiorhodospirales Thiogranum
Ectothiorhodospirales Ectothiorhodospiraceae
Desulfuromonadales Sva1033
Desulfobacterales Desulfobulbus
Desulfobacterales Desulfobulbaceae
D90 D90
Cytophagales Reichenbachiella
Cytophagales Marinoscillum
Cytophagales Fulvivirga
Cytophagales Fabibacter
Cytophagales Cyclobacteriaceae
clade) (SAR406_clade)_Marinimicrobia_(SAR406_clade)_Marinimicrobia_(SAR406_clade)
Chitinophagales Saprospiraceae
Chitinophagales Portibacter
Chitinophagales Lewinella
Chitinophagales Aureispira
Cellvibrionales Sinobacterium
Cellvibrionales SAR92_clade
Cellvibrionales Porticoccus
Cellvibrionales OM60(NOR5)_clade
Cellvibrionales Marimicrobium
Cellvibrionales Luminiphilus
Cellvibrionales Halioglobus
Cellvibrionales Cellvibrionales
Cellvibrionales BD2−7
Cellvibrionales BD1−7_clade
Caulobacterales Robiginitomaculum
Caulobacterales Hellea
Campylobacterales Campylobacterales
Campylobacterales Arcobacter
Betaproteobacteriales RS62_marine_group
Betaproteobacteriales OM43_clade
Betaproteobacteriales IS−44
Betaproteobacteriales Betaproteobacteriales
Bdellovibrionales OM27_clade
BD7−8 BD7−8
Balneolales Balneola
Bacteroidia Bacteroidia
Bacteria Bacteria
B2M28 B2M28
Arenicellales Arenicella
Alteromonadales Psychromonas
Alteromonadales Pseudoalteromonas
Alteromonadales Pseudoalteromonadaceae
Alteromonadales Paraglaciecola
Alteromonadales Moritella
Alteromonadales Colwellia
Alphaproteobacteria Alphaproteobacteria
Actinomarinales Candidatus_Actinomarina
9 9_Subgroup_9_Subgroup_9
Differential Abundance
−4 0 4
Taxa
Timing around Dormancy
coral after
coral before
coral during
water after
water before
water during

cDNA and DNA Core 80% based on sample time

Figure S3

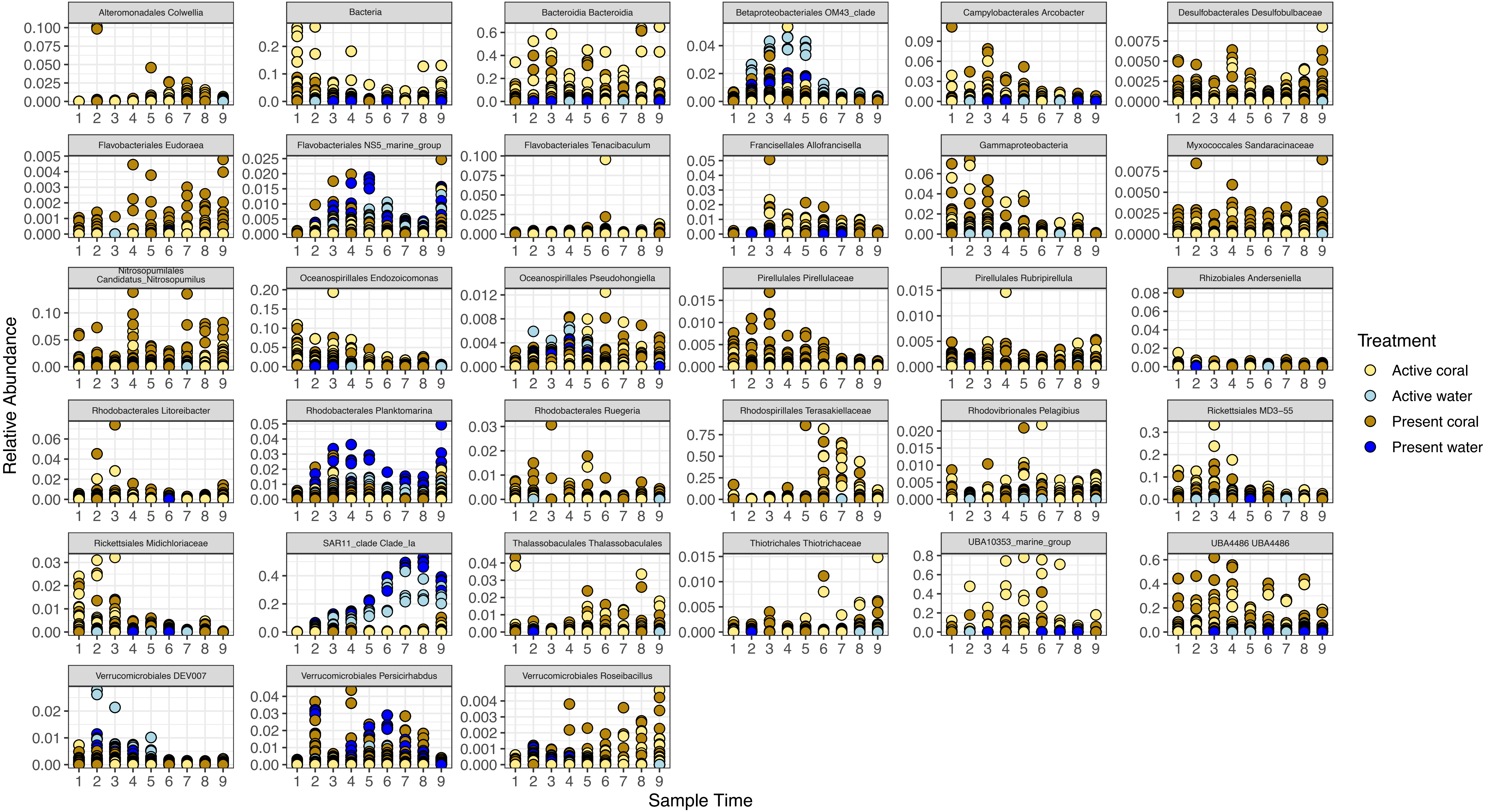

Figure S4

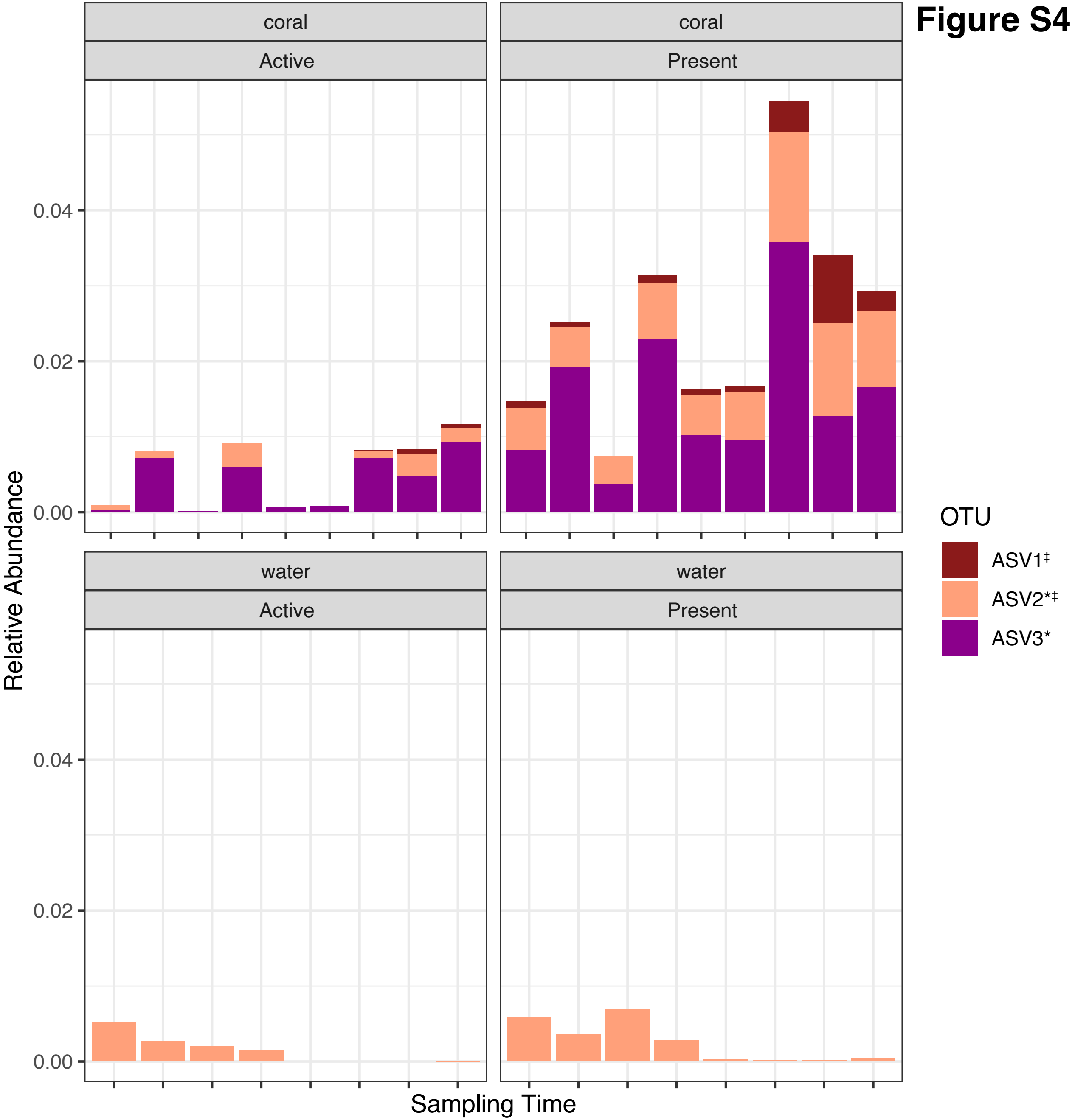

coral
Active
coral
Present
water
Active
water
Present
0.04
0.02
0.00
Relative Abundance
Sampling Time
OTU
ASV1‡
ASV2*‡
ASV3*

Figure S5

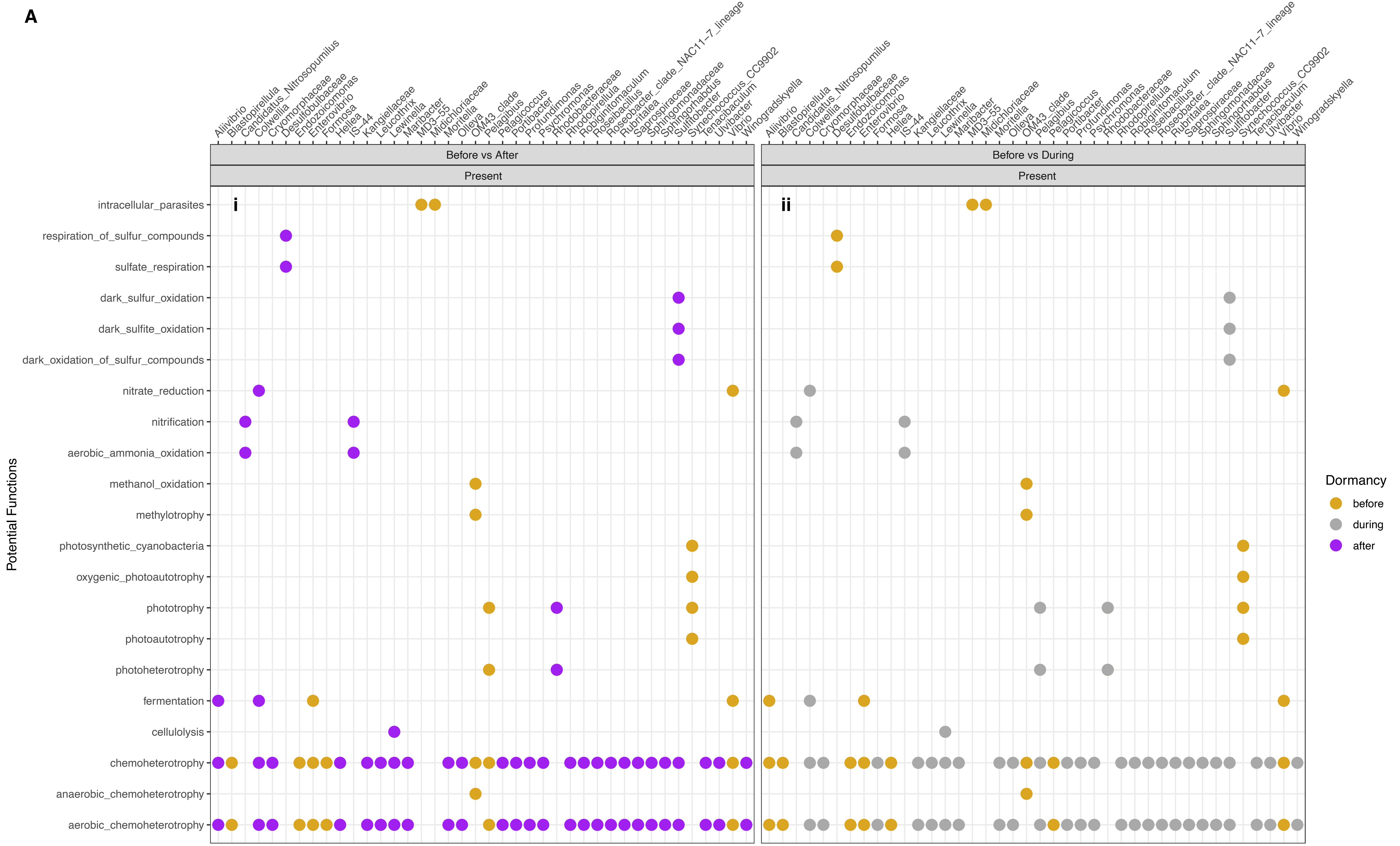
A
Genus
Before vs After
Before vs During
Present
i
ii
Potential Functions
Dormancy
before
during
after
intracellular_parasites
respiration_of_sulfur_compounds
sulfate_respiration
dark_sulfur_oxidation
dark_sulfite_oxidation
dark_oxidation_of_sulfur_compounds
nitrate_reduction
nitrification
aerobic_ammonia_oxidation
methanol_oxidation
methylotrophy
photosynthetic_cyanobacteria
oxygenic_photoautotrophy
phototrophy
photoautotrophy
photoheterotrophy
fermentation
cellulolysis
chemoheterotrophy
anaerobic_chemoheterotrophy
aerobic_chemoheterotrophy
Aliivibrio
Blastopirellula
Candidatus_Nitrosopumilus
Colwellia
Cryomorphaceae
Desulfobulbaceae
Endozoicomonas
Enterovibrio
Formosa
Hellea
IS-44
Kangiellaceae
Leucothrix
Lewinella
Maribacter
MD3-55
Midichloriaceae
Moritella
Olleya
OM43_clade
Pelagibius
Pelagicoccus
Portibacter
Profundimonas
Psychromonas
Rhodobacteraceae
Rhodopirellula
Robiginitomaculum
Roseibacillus
Roseobacter_clade_NAC11-7_lineage
Rubritalea
Saprospiraceae
Sphingomonadaceae
Sphingorhabdus
Sulfitobacter
Synechococcus_CC9902
Tenacibaculum
Ulvibacter
Vibrio
Winogradskyella

Figure S5

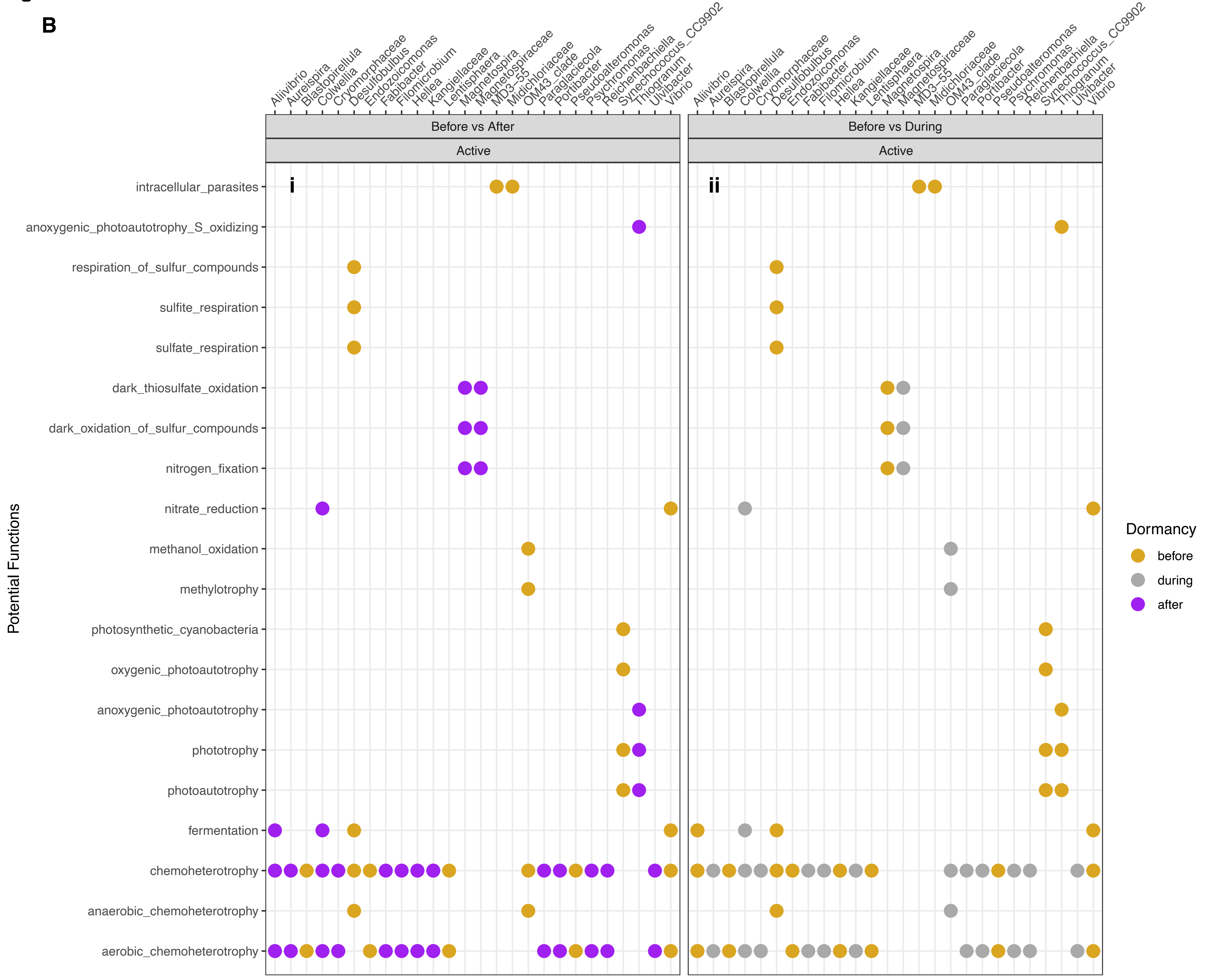

B
Genus
Aliivibrio
Aureispira
Blastopirellula
Colwellia
Cryomorphaceae
Desulfobulbus
Endozoicomonas
Fabibacter
Filomicrobium
Hellea
Kangiellaceae
Lentisphaera
Magnetospira
Magnetospiraceae
MD3-55
Midichloriaceae
OM43_clade
Paraglaciecola
Portibacter
Pseudoalteromonas
Psychromonas
Reichenbachiella
Synechococcus_CC9902
Thiogranum
Ulvibacter
Vibrio
Before vs After
Before vs During
Active
i
ii
Potential Functions
intracellular_parasites
anoxygenic_photoautotrophy_S_oxidizing
respiration_of_sulfur_compounds
sulfite_respiration
sulfate_respiration
dark_thiosulfate_oxidation
dark_oxidation_of_sulfur_compounds
nitrogen_fixation
nitrate_reduction
methanol_oxidation
methylotrophy
photosynthetic_cyanobacteria
oxygenic_photoautotrophy
anoxygenic_photoautotrophy
phototrophy
photoautotrophy
fermentation
chemoheterotrophy
anaerobic_chemoheterotrophy
aerobic_chemoheterotrophy
Dormancy
before
during
after
